# Gender differences and risk factors for smoking among patients with various psychiatric disorders in Saudi Arabia: a cross-sectional study

**DOI:** 10.1186/s13033-018-0201-7

**Published:** 2018-05-03

**Authors:** Fahad D. Alosaimi, Mohammed Abalhassan, Bandar Alhaddad, Ebtihaj O. Fallata, Abdulhadi Alhabbad, Rabab Alshenqiti, Mohammed Z. Alassiry

**Affiliations:** 1 Department of Psychiatry, King Saud University, # 55, King Khalid University Hospital, P.O. Box 7805, Riyadh, 11472 Saudi Arabia; 2 Department of Medicine, Prince Sattam Bin Abdulaziz University, Alkharj, Saudi Arabia; 3 Department of neurosciences, Al-Imam Mohammad Bin Saud University, Riyadh, Saudi Arabia; 4 Mental Health Hospital, Jeddah, Saudi Arabia; 5 Prince Mohammed Medical City, Aljouf, Saudi Arabia; 6 Al-Amal Complex for Mental Health, Dammam, Saudi Arabia; 7 Mental Health Hospital, Abha, Saudi Arabia

**Keywords:** Current smoking, Gender, Psychiatric disorders, Antipsychotics, Saudi Arabia

## Abstract

**Background:**

The higher prevalence of smoking among psychiatric patients is well established. However, gender-specific associations have rarely been the focus of studies among patients with various psychiatric disorders. The aim of this study was to estimate the gender-specific prevalence of current smoking by psychiatric patients and its association with various psychiatric disorders and the use of psychotropic medications.

**Methods:**

A cross-sectional observational study was performed between July 2012 and June 2014. Patients were recruited from six hospitals located in the five regions of Saudi Arabia.

**Results:**

Of the 1193 patients, 402 (33.7%) were current smokers. The incidence of current smoking was much higher among males than females (58.3% versus 6.7%, *p* < 0.001). In one or both genders, current smoking was associated with marital status, education, family income, residence, obesity, physical activity, substance abuse, inpatient status, previous psychiatric hospitalization, and age at onset of psychiatric illness. In both gender, smoking was higher in patients who had a secondary psychiatric disorder (66.7% versus 37.5%, respectively), those who had a primary psychotic disorder (63.7% versus 12.3%), and those taking antipsychotic medication (64.1% versus 8.3%) but lower in patients who had a primary depressive disorder (40.3% versus 4.3%), those who had a primary anxiety disorder (45.8% versus 0.0%), and those taking antidepressant medications (53.7% versus 3.6%). In a multivariate analysis adjusted for demographic/clinical characteristics and psychiatric disorders, current smoking was independently associated with primary psychotic disorders in females (OR = 3.47, 1.45–8.27, *p* = 0.005) but not in males. In a multivariate analysis adjusted for demographic/clinical characteristics and psychotropic medications, current smoking was independently associated with antipsychotic medication use in males (OR = 1.79, 1.10–2.93, *p* = 0.020). Current smoking was strongly associated with substance abuse in both univariate and multivariate analyses.

**Conclusion:**

The prevalence of current smoking is high with marked gender difference in a large sample of mixed psychiatric patients in Saudi Arabia. Smoking-cessation programs may be urgently needed for these vulnerable patients.

## Background

Smoking is a global health risk with a well-established serious morbidity and mortality profile. In 2015, the World Health Organization (WHO) estimated that more than 1 billion people smoke tobacco worldwide, resulting in approximately 6 million deaths per year [1]. In Saudi Arabia, the average prevalence of current tobacco smoking among adults was estimated at 22.6%, with a wide range (11.6–52.3%) reported by studies targeting diverse populations [2]. Interestingly, there is a striking difference in the smoking rates between males (26%) and females (3–9%) in Saudi Arabia [2, 3]. This may partially explain the higher smoking rates found in several studies that included only males, for example, 35% in a military population [4] and 34–52% among male patients at primary care centers [5, 6].

Systematic reviews and other types of reviews have found a positive association between smoking and psychiatric disorders [7, 8], especially among females and in younger population [9]. For example, the prevalence of smoking has been reported to be two- to threefold higher among psychiatric patients than in the general population [10–13]. In addition, smokers have been found to have two- to threefold higher risk of developing psychiatric illness and substance use disorders later in life [14]. Moreover, smoking by psychiatric patients is associated with greater disability and poorer quality of life [15]. The impact of smoking on mortality among psychiatric patients is believed to be even greater than that in the general population, probably because of the excess mortality from cardiovascular diseases, stroke, and respiratory cancers among these patients [16]. The impact on mortality is complicated by the effects of smoking on some psychiatric symptoms, which makes cessation more challenging in these patients [17].

Although a higher smoking prevalence among psychiatric patients is well established, the overall impact is probably affected by several patient factors, including sociodemographic profile, type, and number of psychiatric disorders, pharmacological therapies, and concomitant substance abuse [8, 18–21]. The complex interplay of these factors in different populations complicates comparisons between studies. The objective of the current study was to estimate the prevalence of current smoking in a mixed psychiatric population in Saudi Arabia and examine the effect of the above factors on smoking rates. Suitable data from Saudi Arabia are very limited and outdated, and they lack adjustment for several confounders [22]. In addition, the impact of gender, which has received little attention in Western research, cannot be ignored in our population because of the substantial gender-specific difference in smoking prevalence. Therefore, we examined gender-specific associations between smoking and psychiatric disorders.

## Methods

### Setting

The current study was conducted among patients seeking psychiatric treatment in both inpatient and outpatient psychiatric healthcare services at several hospitals located in the Central, Eastern, Western, Northern, and Southern regions of Saudi Arabia. We aimed to select the largest mental hospital in each of these five major regions. However, because of logistical difficulties, not all the included hospitals were the largest in their region. To compensate, two hospitals were selected from the central region. The final hospitals were King Saud University Medical City in Riyadh and Zulfi General Hospital (Central region), Jeddah Mental Health Hospital (Western region), Al Amal Complex for Mental Health—Dammam (Eastern region), Aljouf Mental Health Hospital (Northern region), and Abha Mental Health Hospital (Southern region). King Saud University Medical City is a university-affiliated governmental hospital, whereas the other hospitals are service based and operate under the authority of the Ministry of Health. All included hospitals provide free psychiatric inpatient and outpatient healthcare services.

### Study design

A cross-sectional observational study was performed between July 2012 and June 2014.

### Population

Consecutive male and female patients seeking psychiatric treatment from the included hospitals over the study period were asked to join the study. Those who provided informed consent were included, irrespective of the type or duration of their psychiatric disorder or recent use of psychotropic medications. Patients whose records and interviews indicated an absence of psychiatric illness (*n* = 59) or lack of smoking status (*N* = 15) were excluded. Therefore, 1193 of the 1264 initial patients were included in the analysis.

### Data collection

A mini-interview form was developed that included sociodemographic characteristics, medical history, current psychiatric disorders, and recent use of psychotropic medications. Data were obtained primarily by reviewing the patients’ charts. The diagnosis of psychiatric disorders in this study was based on routine clinical interviews. The psychiatric consultants in-charge in each study site made psychiatric diagnoses of their patients using the *Diagnostic and Statistical Manual of Mental Disorders*, fourth edition, text revision (DSM-IV-TR) criteria. The psychiatric diagnoses were confirmed by the treating teams, primarily following longitudinal evaluation and follow-up in the psychiatric setting. Unclear or missing information was clarified or obtained by interviewing the patient and/or his or her family. Trained psychiatric residents/staff were in charge for reviewing the chart and conducting the mini-interviews with the patients and/or their families. The purpose of these mini-interviews was to gather any unclear or missing information except for the major clinical data like psychiatric diagnoses and treatments which are certainly documented in the patients’ charts.

The smoking status was determined during the mini-interview using face-to-face questions in Arabic language. Patients were asked to define their cigarette smoking status as a current smoker, a previous smoker (i.e., someone who has smoked greater than 100 cigarettes in their lifetime but has not smoked at least in the last month), or a never-smoker [23].

### Classification of psychiatric disorders

To analyze the data, the psychiatric diagnoses made by the primary psychiatrist for each patient using DSM-IV-TR criteria were classified into 8 categories [24, 25]: *Primary psychotic disorders* included schizophrenia, schizoaffective disorder, delusional disorder, and brief psychotic disorder. *Primary bipolar disorders* included bipolar disorder types I and II. *Primary depressive disorders* included major depressive disorder and dysthymic disorder. *Primary anxiety disorders* included generalized anxiety disorder, obsessive–compulsive disorder, social anxiety disorder, specific phobia, panic disorder, post-traumatic stress disorder, and acute stress disorder. *Personality disorders* included personality disorder not otherwise specified (mixed personality disorder), paranoid personality disorder, antisocial personality disorder, and borderline personality disorder. *Secondary psychiatric disorders* included psychotic disorder due to another medical condition, depression due to another medical condition, dementia, substance abuse, and substance-induced depressive disorder. *Other disorders* included undifferentiated somatoform disorder, conversion disorder, mental retardation, attention deficit hyperactivity disorder, dissociative disorder, primary insomnia, adjustment disorder, enuresis disorder, trichotillomania, and anorexia nervosa. *Multiple disorders* included two or more of the psychiatric illnesses listed above.

### Classification of psychotropic medications

Both individual psychotropic medications and pharmacologic groups were used in the analysis. These included antipsychotics (low potency first generation, high potency first generation, and second generation), antidepressants (e.g., selective serotonin reuptake inhibitors (SSRIs) and tricyclics), mood stabilizers, and antianxiety medications.

### Statistical analysis

Categorical data are presented using frequencies and percentages, and continuous data are presented using means and standard deviations (SD). Because of the marked difference in smoking prevalence by gender, analysis was completed separately for males and females. As there were very few differences between previous and never-smokers as regards psychiatric disorders and use of psychotropic medications, patients were categorized as either current smokers or non-current smokers (including previous and never-smokers) to simplify data interpretation. Significant differences between current and non-current smokers with regard to demographics, clinical characteristics, disorders, and medications were tested. Categorical data were tested using the *χ*^2^ test or Fisher’s exact test (as appropriate), and continuous data were tested using Student’s *t* test or the Mann–Whitney *U* test (as appropriate). Independent associations of current smoking status with different psychiatric disorders and psychotropic medications, after adjusting for relevant demographic and clinical characteristics, were evaluated using multivariate logistic regression models with stepwise backward elimination. Smoking in small-sized groups with sample size < 20 or with zero smoking prevalence (as those with hypothyroidism) were excluded from the model to avoid instability (very huge or zero odds ratio). All *p* values were two-tailed. *p* < 0.05 was considered significant. SPSS software (release 20.3, Armonk, NY: IBM Corp) was used for all statistical analyses.

## Results

### Prevalence of current smoking

**Fig. 1:**
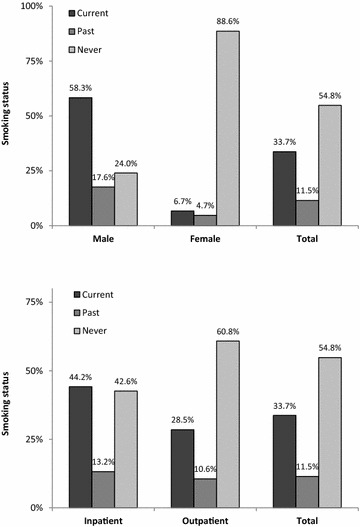
Smoking status by gender (top) and type of patient (below) among psychiatric patients (*N* = 1193). *p < 0.001 for each statistic

A total of 1193 patients (624 males and 569 females) were included in the analysis; 799 (67.0%) outpatients and 394 (33.0%) inpatients. Of the total group, 402 (33.7%) patients were current smokers, 137 (11.5%) were previous smokers, and 654 (54.8%) were never-smokers. The prevalence of smoking, either currently or previously, was much higher among males (58.3 and 17.6%, respectively) than females (6.7 and 4.7%, respectively), and the difference was statistically significant (*p* < 0.001). In addition, the prevalence of current smokers was significantly higher among inpatients than among outpatients (44.2% versus 28.5%, *p* < 0.001, Fig. 1).

### Demographic characteristics and smoking

The average age was 38.0 ± 13.0 years, and more than half (54.3%) of the patients were unmarried (Table 1). The majority were either illiterate or had less than a secondary education (87.1%), were non-working (71.2%), had a monthly family income of 6000 SR or less (62.0%), and were living in urban communities (80.4%). Approximately 37.9% of the patients were obese, and the majority (87.3%) were physically inactive. The sociodemographic characteristics associated with current smoking varied by gender. In males, higher rates of current smoking (average 58.3%) were significantly associated with being unmarried (*p* < 0.001), having a secondary education or less (*p* < 0.001), having lower family income (*p* = 0.009), living in a village (*p* = 0.004), and being non-obese (*p* = 0.010). In females, higher rates of current smoking (average 6.7%) were significantly associated with having a secondary education or less (*p* = 0.010) and being physically active (*p* = 0.017, Table 1).

**Table 1:** Demographic characteristics by gender and current smoking status among psychiatric patients (*N* = 1193)

	Overall (*N* = 1193)	Current smoking in males (*N* = 624)			Current smoking in females (*N* = 569)		
		No (*N* = 260)	Yes (*N* = 364)	*p* value	No (*N* = 531)	Yes (*N* = 38)	*p* value
Age (years)							
Mean ± SD	38.0 ± 13.0	37.1 ± 14.0	37.8 ± 11.2	0.516	38.7 ± 13.8	36.5 ± 9.9	0.331
< 40	708 (59.6%)	163 (41.5%)	230 (58.5%)	0.081	293 (93.0%)	22 (7.0%)	0.329
40–60	420 (35.4%)	76 (38.4%)	122 (61.6%)		207 (93.2%)	15 (6.8%)	
> 60	60 (5.1%)	18 (60.0%)	12 (40.0%)		30 (100.0%)	0 (0.0%)	
Marital status							
Married	524 (45.7%)	107 (49.8%)	108 (50.2%)	< 0.001	293 (94.8%)	16 (5.2%)	0.359
Single	504 (44.0%)	125 (37.5%)	208 (62.5%)		157 (91.8%)	14 (8.2%)	
Divorced	106 (9.2%)	8 (19.0%)	34 (81.0%)		58 (90.6%)	6 (9.4%)	
Widowed	12 (1.0%)	0 (0.0%)	0 (0.0%)		12 (100.0%)	0 (0.0%)	
Number of children	3.5 ± 3.2	3.9 ± 3.6	3.3 ± 2.9	0.180	3.5 ± 3.2	2.9 ± 3.1	0.374
Educational level							
Illiterate	251 (21.5%)	45 (55.6%)	36 (44.4%)	< 0.001	165 (97.1%)	5 (2.9%)	0.010
Secondary or less	766 (65.6%)	161 (35.8%)	289 (64.2%)		287 (90.8%)	29 (9.2%)	
University/other	151 (12.9%)	46 (61.3%)	29 (38.7%)		74 (97.4%)	2 (2.6%)	
Working status							
Not working	832 (71.2%)	151 (42.5%)	204 (57.5%)	0.492	450 (94.3%)	27 (5.7%)	0.301
Working	336 (28.8%)	103 (39.8%)	156 (60.2%)		70 (90.9%)	7 (9.1%)	
Family income (SR)							
≤ 3000	344 (29.8%)	73 (38.2%)	118 (61.8%)	0.009	143 (93.5%)	10 (6.5%)	0.695
3001–6000	372 (32.2%)	72 (35.5%)	131 (64.5%)		161 (95.3%)	8 (4.7%)	
6001–9000	241 (20.8%)	53 (44.2%)	67 (55.8%)		112 (92.6%)	9 (7.4%)	
> 9000	199 (17.2%)	48 (55.8%)	38 (44.2%)		104 (92.0%)	9 (8.0%)	
Residential type							
City	955 (80.4%)	225 (43.1%)	297 (56.9%)	0.004	403 (93.1%)	30 (6.9%)	0.411
Village	206 (17.3%)	29 (30.9%)	65 (69.1%)		106 (94.6%)	6 (5.4%)	
Desert	27 (2.3%)	6 (85.7%)	1 (14.3%)		20 (100.0%)	0 (0.0%)	
Body mass index (BMI)							
Mean ± SD	28.7 ± 7.7	28.4 ± 6.5	27.4 ± 7.1	0.096	29.8 ± 8.4	29.3 ± 8.2	0.738
Non-obese	719 (62.1%)	161 (38.0%)	263 (62.0%)	0.010	273 (92.5%)	22 (7.5%)	0.218
Obese	438 (37.9%)	94 (49.0%)	98 (51.0%)		234 (95.1%)	12 (4.9%)	
Physical activity							
Active	149 (12.7%)	40 (42.1%)	55 (57.9%)	0.920	46 (85.2%)	8 (14.8%)	0.017
Not active	1028 (87.3%)	214 (41.6%)	301 (58.4%)		484 (94.3%)	29 (5.7%)	

### Clinical characteristics and smoking

The average age at onset of psychiatric illness was 28.3 ± 12.0 years, with average illness duration of 9.7 ± 9.3 years (Table 2). Approximately 24.4% of the patients have substance abuse disorder. Almost half (46.0%) had had an average of 3.8 ± 4.4 previous psychiatric hospitalizations. Common comorbidities included diabetes and hypertension. In both males and females, higher rates of current smoking were significantly associated with substance abuse (*p* < 0.001 each), inpatient status (*p* = 0.001 and *p* = 0.041, respectively), and previous psychiatric hospitalization (*p* < 0.001 and *p* = 0.004, respectively), as well as—in females only—a younger age at onset of psychiatric illness (*p* = 0.039, Table 2).

**Table 2:** Clinical characteristics by current smoking status among psychiatric patients (*N* = 1193)

	Overall (*N* = 1193)	Current smoking in males (*N* = 624)			Current smoking in females (*N* = 569)		
		No (*N* = 260)	Yes (*N* = 364)	*p* value	No (*N* = 260)	Yes (*N* = 364)	*p* value
Hospitalization status							
Inpatient	394 (33.0%)	78 (33.1%)	158 (66.9%)	0.001	142 (89.9%)	16 (10.1%)	0.041
Outpatient	799 (67.0%)	182 (46.9%)	206 (53.1%)		389 (94.6%)	22 (5.4%)	
Age at disease onset							
Mean ± SD	28.3 ± 12.0	27.4 ± 12.1	28.1 ± 10.0	0.466	29.2 ± 13.2	24.6 ± 9.6	0.039
< 25 years	487 (41.6%)	120 (46.3%)	139 (53.7%)	0.059	209 (91.7%)	19 (8.3%)	0.179
≥ 25 years	684 (58.4%)	137 (38.7%)	217 (61.3%)		312 (94.5%)	18 (5.5%)	
Disease duration							
Mean ± SD	9.7 ± 9.3	9.7 ± 9.6	9.6 ± 8.0	0.872	9.5 ± 10.0	11.9 ± 10.5	0.161
≤ 10 years	781 (66.8%)	175 (42.5%)	237 (57.5%)	0.593	349 (94.6%)	20 (5.4%)	0.108
> 10 years	388 (33.2%)	80 (40.2%)	119 (59.8%)		172 (91.0%)	17 (9.0%)	
Previous psychiatric hospitalization							
No	644 (54.0%)	151 (49.2%)	156 (50.8%)	< 0.001	323 (95.8%)	14 (4.2%)	0.004
Yes	549 (46.0%)	109 (34.4%)	208 (65.6%)		208 (89.7%)	24 (10.3%)	
Mean ± SD	3.8 ± 4.4	2.8 ± 3.3	4.1 ± 5.1	0.019	4.0 ± 4.0	3.7 ± 4.0	0.704
Medical history							
Diabetes mellitus	113 (9.5%)	26 (47.3%)	29 (52.7%)	0.377	56 (96.6%)	2 (3.4%)	0.411
Hypertension	92 (7.7%)	16 (30.2%)	37 (69.8%)	0.076	37 (94.9%)	2 (5.1%)	> 0.95
Hyperthyroidism	25 (2.1%)	1 (50.0%)	1 (50.0%)	> 0.95	22 (95.7%)	1 (4.3%)	> 0.95
Hypothyroidism	39 (3.3%)	4 (100.0%)	0 (0.0%)	0.029	34 (97.1%)	1 (2.9%)	0.714
Cerebrovascular accident	14 (1.2%)	3 (50.0%)	3 (50.0%)	0.697	7 (87.5%)	1 (12.5%)	0.427
Hyperlipidemia	12 (1.0%)	3 (33.3%)	6 (66.7%)	0.742	3 (100.0%)	0 (0.0%)	> 0.95
Epilepsy	12 (1.0%)	4 (66.7%)	2 (33.3%)	0.241	5 (83.3%)	1 (16.7%)	0.341
Kidney disease	10 (0.9%)	5 (100.0%)	0 (0.0%)	0.012	5 (100.0%)	0 (0.0%)	> 0.95
Asthma	8 (0.7%)	4 (66.7%)	2 (33.3%)	0.241	2 (100.0%)	0 (0.0%)	> 0.95
Substance abuse	273 (24.4%)	52 (21.3%)	192 (78.7%)	< 0.001	22 (75.9%)	7 (24.1%)	< 0.001
Medication use							
Diabetes	83 (7.0%)	19 (45.2%)	23 (54.8%)	0.627	39 (95.1%)	2 (4.9%)	> 0.95
Hypertension	44 (3.7%)	12 (40.0%)	18 (60.0%)	0.849	14 (100.0%)	0 (0.0%)	0.614
Hyperlipidemia	19 (1.6%)	6 (46.2%)	7 (53.8%)	0.740	6 (100.0%)	0 (0.0%)	> 0.95

### Psychiatric disorders/medications and smoking

The majority of patients had a single psychiatric disorder (90.4%): mainly primary psychotic disorders (41.2%), primary depressive disorders (16.8%), and primary bipolar disorders (15.3%, Table 3). The majority of patients were using two or more psychotropic medications (66.5%): primarily antipsychotics (76.7%), antidepressants (41.6%), and mood stabilizers (27.8%). For both males and females, higher rates of current smoking were positively associated with primary psychotic disorders (*p* = 0.004 and *p* = 0.001, respectively) and antipsychotic medications (*p* < 0.001 and *p* = 0.004, respectively) but negatively associated with primary anxiety disorders (*p* = 0.040 and *p* = 0.009, respectively) and antidepressant medications (*p* = 0.021 and *p* = 0.012, respectively). In addition, higher rates of current smoking were positively associated with the use of multiple psychotropic medications in males (*p* = 0.004) and secondary psychiatric disorders in females (*p* = 0.012) but negatively associated with primary depressive disorders in males (*p* = 0.002, Table 3).

**Table 3:** Psychiatric disorders and use of psychotropic medications by current smoking status

	Overall (*N* = 1193)	Current smoking in males (*N* = 624)			Current smoking in females (*N* = 569)		
		No (*N* = 260)	Yes (*N* = 364)	*p* value	No (*N* = 260)	Yes (*N* = 364)	*p* value
Number of psychiatric disorders							
One	1079 (90.4%)	233 (41.5%)	329 (58.5%)	0.751	481 (93.0%)	36 (7.0%)	0.391
Two or more	114 (9.6%)	27 (43.5%)	35 (56.5%)		50 (96.2%)	2 (3.8%)	
Psychiatric disorders							
Primary psychotic disorders	491 (41.2%)	119 (36.3%)	209 (63.7%)	0.004	143 (87.7%)	20 (12.3%)	0.001
Primary bipolar disorders	183 (15.3%)	29 (41.4%)	41 (58.6%)	0.966	106 (93.8%)	7 (6.2%)	0.818
Primary depressive disorders	200 (16.8%)	37 (59.7%)	25 (40.3%)	0.002	132 (95.7%)	6 (4.3%)	0.208
Primary anxiety disorders	131 (11.0%)	32 (54.2%)	27 (45.8%)	0.040	72 (100.0%)	0 (0.0%)	0.009
Personality disorders	18 (1.5%)	1 (12.5%)	7 (87.5%)	0.148	10 (100.0%)	0 (0.0%)	> 0.95
Secondary psychiatric disorders	44 (3.7%)	12 (33.3%)	24 (66.7%)	0.296	5 (62.5%)	3 (37.5%)	0.012
Multiple disorders	80 (6.7%)	15 (35.7%)	27 (64.3%)	0.418	36 (94.7%)	2 (5.3%)	> 0.95
Other disorders	46 (3.9%)	15 (78.9%)	4 (21.1%)	0.001	27 (100.0%)	0 (0.0%)	0.245
Number of psychotropic medications							
One	363 (33.5%)	97 (47.8%)	106 (52.2%)	0.004	148 (92.5%)	12 (7.5%)	0.497
Two or more	719 (66.5%)	129 (35.4%)	235 (64.6%)		334 (94.1%)	21 (5.9%)	
Psychotropic medications							
Any antipsychotic	830 (76.7%)	164 (35.9%)	293 (64.1%)	< 0.001	342 (91.7%)	31 (8.3%)	0.004
First generation, low potency	59 (5.5%)	11 (42.3%)	15 (57.7%)	0.794	32 (97.0%)	1 (3.0%)	0.713
First generation, high potency	177 (16.4%)	44 (41.9%)	61 (58.1%)	0.635	66 (91.7%)	6 (8.3%)	0.440
Second generation	733 (67.7%)	147 (35.7%)	265 (64.3%)	0.001	292 (91.0%)	29 (9.0%)	0.002
Any antidepressant	450 (41.6%)	93 (46.3%)	108 (53.7%)	0.021	240 (96.4%)	9 (3.6%)	0.012
SSRI antidepressants	312 (28.8%)	63 (50.4%)	62 (49.6%)	0.006	180 (96.3%)	7 (3.7%)	0.062
Tricyclic antidepressants	69 (6.4%)	10 (47.6%)	11 (52.4%)	0.459	45 (93.8%)	3 (6.3%)	> 0.95
Other antidepressants	158 (14.6%)	28 (32.6%)	58 (67.4%)	0.133	70 (97.2%)	2 (2.8%)	0.133
Mood stabilizers	301 (27.8%)	59 (38.6%)	94 (61.4%)	0.701	140 (94.6%)	8 (5.4%)	0.555
Antianxiety	68 (6.3%)	8 (29.6%)	19 (70.4%)	0.266	39 (95.1%)	2 (4.9%)	> 0.95

**Fig. 2:**
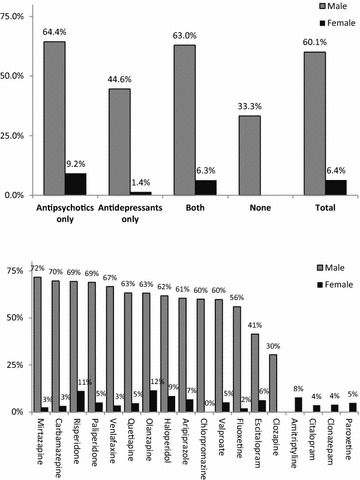
Prevalence of current smoking by the use of group (above) and individual (below) psychotropic medications among psychiatric patients (*N* = 1193). *p value for the upper panel is 0.001 for males and 0.015 for females. **Drugs used by fewer than 20 male or female patients were excluded

In both males and females, the prevalence of current smoking was higher among patients using antipsychotic medications alone or combined with antidepressant medications and was the lowest among those not using any psychotropic medication or using antidepressant medications alone (*p* = 0.001 and *p* = 0.015, respectively, Fig. 2). At least two-thirds of male patients using mirtazapine, carbamazepine, risperidone, paliperidone, or venlafaxine were current smokers. Moreover, risperidone and olanzapine were associated with a higher prevalence of current smoking in females.

### Independent associations of smoking

Multivariate logistic regression analysis models tested the association of current smoking with different psychiatric disorders and the use of different psychotropic medications after adjusting for significant gender-specific demographic, clinical, and behavioral characteristics (Tables 1 and 2). They included marital status, educational level, family income, type of residential area, obesity, physical activity, hospitalization status, previous psychiatric hospitalization, and substance abuse (Table 4). In models additionally adjusted for psychiatric disorders, but not for psychotropic medications, current smoking was independently associated with primary psychotic disorders in females (OR = 3.47, 1.45–8.27, *p* = 0.005), but not in males, and with substance abuse in both males (OR = 5.28, 3.52–7.93, *p* < 0.001) and females (OR = 10.56, 3.78–29.53, *p* < 0.001). In addition, education and lower family income were independently associated with current smoking in males. In models additionally adjusted for psychotropic medications but not psychiatric disorders, current smoking was independently associated with antipsychotic medications in males (OR = 1.79, 1.10–2.93, *p* = 0.020) and marginally associated in females (OR = 4.68, 0.95–22.95, *p* = 0.057) and with substance abuse in both males (OR = 5.18, 3.39–7.92, *p* < 0.001) and females (OR = 14.15, 4.32–46.35, *p* < 0.001). In addition, lower family income was independently associated with current smoking in females (Table 4).

**Table 4:** Multivariate logistic regression analysis for the associations of current smoking among male and female patients among psychiatric patients (*N* = 1193)

	Current smoking in males (*N* = 543)			Current smoking in females (*N* = 505)		
	Odds ratio	95% confidence interval	*p* value	Odds ratio	95% confidence interval	*p* value
Full adjustment^a^ plus disorders						
Education (educated)	*1.82*	*1.03–3.23*	*0.040*			
Family income (< 6000 SR)	*1.90*	*1.26–2.86*	*0.002*			
BMI (non-obese)	1.49	1.00–2.22	0.053			
Hospitalization status (inpatient)	*1.72*	*1.16–2.56*	*0.007*			
Substance abuse	*5.28*	*3.52–7.93*	*< 0.001*	*10.56*	*3.78–29.53*	*< 0.001*
Primary psychotic disorders				*3.47*	*1.45–8.27*	*0.005*
Full adjustment^a^ plus medications						
Education (educated)				3.09	0.90–10.55	0.072
Marital status (unmarried)	1.44	0.94–2.20	0.092			
Family income (< 6000 SR)	*1.58*	*1.04–2.39*	*0.032*			
Hospitalization status (inpatient)				2.38	0.91–6.20	0.077
Age at disease onset (≥ 25 years)				*2.94*	*1.00–8.46*	*0.049*
Substance abuse	*5.18*	*3.39–7.92*	*< 0.001*	*14.15*	*4.32–46.35*	*< 0.001*
Antipsychotic medications	*1.79*	*1.10–2.93*	*0.020*	4.68	0.95–22.95	0.057

Data in italic means it is statistically significant (*p* value is < 0.05)

^a^Adjusted for the following in males: marital status, educational level, family income, type of residential area, obesity, hospitalization status, previous psychiatric hospitalization, and substance abuse. Adjusted for the following in females: educational level, physical activity, hospitalization status, age at disease onset, previous psychiatric hospitalization, and substance abuse

## Discussion

We found a high prevalence of current smoking in a large sample of psychiatric outpatients and inpatients in Saudi Arabia. As in the general Saudi population—although at a higher rate—smoking prevalence was much higher among males than females (58% versus 7%). This was almost double the rate of current smoking recently reported by the WHO for the general Saudi population (26% in males and 3% in females) [3]. However, the prevalence we found was almost identical to the current smoking rates (58%) reported among 505 male outpatients recruited from five hospitals in Saudi Arabia in 1996 and 1997 [22]. Unfortunately, females were not included in the previous study, which reported the only published data regarding smoking by psychiatric patients in Saudi Arabia [22]. Several hypotheses have been suggested to explain the higher smoking rates among psychiatric patients [8, 26]. The most widely accepted hypothesis is self-medication; that is, patients smoke to reduce the impact of specific psychiatric disorders and medications [8, 17, 20, 26]. Other hypotheses include shared genetic factors that control the susceptibility for both smoking and psychiatric disorders and shared environmental factors, such as stress, that can increase the incidence of both smoking and psychiatric illness [8, 26].

Higher rates of current smoking among male psychiatric patients is consistent with the findings of nearly all Western and Eastern studies [20, 27–32]. For example, male versus female rates have been reported as 66% versus 56% in the US [32], 69% versus 48% in France [20], 32% versus 21% in Japan [29], 59% versus 41% in Brazil [28], and 78.4% versus 36.2% in Iran [30]. However, a very large male–female difference, as found in the current study, has been reported in only a few Eastern countries, such as India (53% versus 9%) [31] and Bahrain (47% versus 4%) [27]. This reflects the variability in gender-specific rates of current smoking in the general population of Western and Eastern countries [33]. Smoking among females in a conservative society such as Saudi Arabia could be perceived as impairing the feminine Islamic image of women [2, 34]. Another explanation is that it is more inconvenient for females to obtain cigarettes and find a place to smoke [34]. However, we cannot exclude the possibility that current smoking by female patients is under-reported as a consequence of its social stigma [2]. Interestingly, the persistence of a marked gender difference in smoking rates among our patients, after stratifying by psychiatric disorder and psychotropic medication, might indicate that the gender effect is independent of the psychiatric impact. In addition, the persistence of sociodemographic risk factors for smoking (such as low education, low income, and being unmarried) in a multivariate analysis, especially in men, is more evidence that the motivations for smoking in the general population [4, 35] are the same as those in psychiatric patients [5, 36, 37]. The disappearance/attenuation of sociodemographic risk factors for smoking in multivariate analysis among females could be related to the low prevalence of smoking among this group rather than real lack of associations.

As found in previous studies, smoking rates in the current study were highest among patients with substance abuse disorder [26, 29]. In addition, current smoking and substance abuse were strongly and independently associated, even after adjusting for psychiatric disorder or psychotropic medication [36, 37]. This may further explain the high smoking prevalence among those with secondary psychiatric disorders in the current and previous studies [20, 22, 28]. A large twin study showed that shared genetic and, to a lesser extent, environmental risk factors predispose individuals to lifetime comorbidity of common psychiatric and substance abuse disorders [38].

Although attenuated in multivariate analyses, smoking in the current study was strongly associated with primary psychotic disorders and/or use of antipsychotic medications. High smoking rates among patients with schizophrenia/schizoaffective disorders have been consistently reported in previous studies: 66–74% in previous studies [12, 18, 20, 22, 32, 36] compared to 64% in the current study. Higher smoking rates were also reported among patients using antipsychotic medications [20]. It has been suggested that smoking reduces the intensity of some psychiatric symptoms and improves cognitive and concentration functions in patients with schizophrenia [8, 39, 40]. However, smoking is also associated with an increased risk of psychosis and an earlier age at onset of psychotic illness [41]. In addition, smoking was reported to reduce the side effects of antipsychotic medications, especially extrapyramidal manifestations [40, 42]. Yet, smoking can have effects on the metabolism of antipsychotic medications, especially clozapine and olanzapine, such as decreasing their blood levels and increasing the clinically effective dose [43]. Therefore, it has been suggested that doses of clozapine and olanzapine be increased by 50 and 30%, respectively, for smokers to attain an equivalent clozapine or olanzapine blood concentration [44]. The complex association between smoking and schizophrenia, as well as antipsychotic medications, can make smoking cessation challenging and cause sudden withdrawal symptoms [17, 43]. Interestingly, the lower smoking rates among patients with primary depressive disorders and/or those using antidepressant medications, which were determined in univariate analysis in the current study, have been previously reported [20, 30, 31, 36]. Although it may be difficult to explain, the fact that the majority of our patients who were not using antidepressants were using antipsychotics may partially explain the relatively lower smoking rates among patients using antidepressant medications. In addition, the apparently beneficial effects of smoking observed in patients treated with antipsychotics are lacking among patients treated with antidepressants. In the other hand, except of bupropion and nortriptyline, evidence suggests that most antidepressants has no antismoking effect [45].

Several clinical guidelines recommend giving higher priority to providing smoking-cessation advice in everyday clinical practice to patients with a mental health diagnosis [46–48]. A recent systematic review found that people with severe mental illness receive smoking-cessation advice about as often as people without mental illness do, whereas those with non-severe mental illness are slightly more likely to receive smoking-cessation advice compared with people without mental illness [48]. In addition to the benefits of smoking cessation in decreasing morbidity and mortality in people with psychiatric disorders [46], smoking cessation is also associated with reduced depression, anxiety, and stress, and improved positive mood and quality of life to the same extent for those with psychiatric disorders as for those without [49]. The big positive effect-size after smoking cessation in both those with or without psychiatric disorders may indicate the common personal and social motives of smoking in both groups [49]. However, traditional smoking-cessation programs may need to be tailored to fit the neuropsychological profile of psychiatric patients [50]. The European Psychiatric Association provides seven recommendations regarding the core components of the diagnostics and treatment of tobacco dependence in adults with mental illness [46]: (1) the process for recording smoking status, (2) the timing of the intervention, (3) counseling, (4) drug treatment using a first-line product (e.g., nicotine replacement therapy, varenicline, bupropion), (5) contacting the patient soon after cessation, (6) follow-up visits, and (7) relapse prevention and management [46].

The current study makes several important contributions. It is the first study in Saudi Arabia to estimate the gender-specific prevalence of current smoking and to determine its independent risk factors among male and female psychiatric patients. The mixed population and the relatively large sample size enabled us to examine the association of current smoking with a wide variety of psychiatric disorders and psychotropic medications. Previous studies focused primarily on antipsychotics and rarely on the variability of smoking rates among patients taking different groups of psychotropic medications. However, we acknowledge several limitations. First, the use of convenience sampling may limit the generalizability of our findings to psychiatric patients in Saudi Arabia. The cross-sectional design did not enable us to ascertain causality between psychiatric disorders or psychotropic medications and current smoking. Finally, although cigarette smoking is by far the most common type of smoking in Saudi Arabia, the findings of the current study do not reflect other types of tobacco smoking.

## Conclusion

We found a high prevalence of current smoking among a large sample of psychiatric patients in Saudi Arabia. As in the general population, there is a striking gender difference in smoking rates. In addition to sociodemographic risk factors for smoking, substance abuse, psychotic disorders, and antipsychotic medications are strong independent associates. The current findings suggest an urgent need for smoking-cessation programs for these vulnerable patients as well as for further studies to test the effectiveness of such programs.

## Abbreviations

OR: odds ratio
WHO: World Health Organization
DSM-IV-TR: Diagnostic and Statistical Manual of Mental Disorders, fourth edition, text revision
